# Characterization of Seeding Conditions for Studies on Differentiation Patterns of Subventricular Zone Derived Neurospheres

**DOI:** 10.3389/fncel.2016.00055

**Published:** 2016-03-07

**Authors:** Eduardo H. Sanchez-Mendoza, Jana Schlechter, Dirk M. Hermann, Thorsten R. Doeppner

**Affiliations:** ^1^Department of Neurology, Essen Medical School, University of Duisburg-EssenEssen, Germany; ^2^Department of Neurology, Medical School, University of GöttingenGöttingen, Germany

**Keywords:** stem cells, neural progenitor cells, neurospheres, cell differentiation, PDL, PornT, cell culture

## Abstract

Stem cell research depends on extensive *in vitro* research. Poly-D-lysine (PDL) and polyornithine (PornT) are chemically synthesized amino acid chains promoting cell adhesion to solid substrates. Although, PDL and PornT are extensively used, there is no common agreement regarding the most optimal substance and its concentration. We therefore aimed at testing the effect of increasing concentrations (10, 50, and 100 μg/ml) for each compound and their corresponding mixtures (5+5 and 10+10 μg/ml) on the differentiation patterns of subventricular zone derived neurospheres. The latter were cultured for 24 h for protein and morphological analysis or for 8 h for migration analysis. No significant differences were found between increasing concentrations of PDL and PornT alone and the 10+10 condition in Western blots and immunocytochemistry. However, the mixed condition of 5+5 showed decreased glial fibrillary acidic protein and nestin expression with no changes in Akt, pAkt, GSK-3-beta, and pGSK-3-beta expression patterns. The various coating conditions also had no influence on migration of cells emerging from the neurosphere. Nevertheless, stimulation with recombinant human Erythropoietin (rhEpo) reduced migration by 20% regardless of the coating condition. We therefore conclude that a minimal concentration of 10 μg/ml of either compound should be used to produce reliable results with no alterations in protein levels as found for the 5+5 groups, and that the coating has no effect on the response of cells to chemical interventions. As such, a concentration of 10 μg/ml for either substance is sufficient when studying cellular processes of neurospheres in an *in vitro* or *ex vivo* environment.

## Introduction

Poly-D-lysine (PDL) and polyornithine (PornT) are non-specific attachment factors that promote cell adhesion to solid substrates. They are the two most often used coating materials for *in vitro* or *ex vivo* experiments and essential for the study of cellular characteristics and processes (Greilberger et al., 1997; Ramsay and Gumbleton, 2002; Louis and Reynolds, 2005; Lai et al., 2008; Ahlenius et al., 2009; Yang et al., 2015). As *in vitro* experiments are merely simplified replica of complex cellular *in vivo* networks, the artificial environment created in an *in vitro* experiment needs to make use of enrichment factors. To ensure proper cell adhesion and therefore the course of the experiment, it is important to choose the best coating factor possible. An important advantage of PDL and PornT is that they are artificially produced amino acid chains and are thus thought to be resistant to enzymatic degradation. This prolongs cell adhesion compared to naturally occurring compounds like poly-L-lysine (PLL; Mazia et al., 1975). Due to this and their easy availability, both PDL and PornT have already extensively been used for *in vitro* experiments with neural progenitor cells (NPCs) and/or neurospheres (Wachs et al., 2003; Lee et al., 2005; Chojnacki and Weiss, 2008; Ahlenius et al., 2009; Ladiwala et al., 2012). These types of cells are readily produced and have the ability to differentiate into astrocytes, neurons, and oligodendrocytes (Chojnacki and Weiss, 2008).

To study differentiation mechanisms in neurospheres, there are several known cellular markers. Nestin is an intermediate filament protein and is expressed in dividing cells. An important characteristic is that nestin does not persist during adulthood. Its expression will faint during the transition from neurosphere to neuron (Romero-Ramos et al., 2002). Other intermediate filaments will take over. One example for a cell-type specific intermediate filament is glial fibrillary acidic protein (GFAP), which is primarily expressed by astrocytes (Sofroniew and Vinters, 2010). These transitional states can be investigated by their underlying molecular mechanisms. Akt, GSK3-β and both their activated phosphorylated forms play important roles during these phases (Shioda et al., 2009; Rahmani et al., 2013). The activation of Akt through phosphorylation leads to activation of downstream targets like mTOR and VEGF to increase cell proliferation, cell growth, and protein synthesis in neurons (Peltier et al., 2007; Thau-Zuchman et al., 2010; Kitagishi et al., 2012). Furthermore, the Akt activation results in inhibition of GSK3, which in turn inhibits β-catenin. With the inhibition of GSK3 due to phosphorylation, β-catenin is increased and can activate downstream mechanisms, which lead to the induction of neurogenesis and angiogenesis (Fang et al., 2000; Tsujio et al., 2000; Endo et al., 2006; Hur and Zhou, 2010).

There is no broad consensus whether or not differentiation rates of cultured neurospheres are affected when using either PDL or PornT. Another issue that needs to be dealt with focuses on the optimal concentration to be used, which seems to depend on investigator’s preferences only. We therefore aimed at investigating which coating compound (PDL vs. PornT) and which concentration is more suitable to induce proper cellular differentiation and the possible influence of the coating on the response to chemical interventions.

## Materials and Methods

### Animals

The present study protocol was approved by and was performed according to the guidelines of local authorities (Bezirksregierung Duesseldorf, TSG966/08). C57BL6 mice, obtained from Harlan Netherlands, were housed at a 12-h circadian rhythm and fed *ad libitum*. Offspring were used for neurosphere preparation.

### Preparation of Neural Progenitor Cells

Neurospheres were obtained from postnatal days 3–6 (P3–P6) pups, which were sacrificed by decapitation. Brains were incubated in dissection medium [1% penicillin/streptomycin (P/S) and 99% Hank’s balanced salt solution (HBSS)]. Thereafter, the subventricular zone (SVZ) was extracted and the tissue was incubated in a 0.05% trypsin solution followed by incubation for 10 min at 37°C. The tissue was rinsed with dissection medium three times and 1 ml of culture media (DMEM/F-12+ GlutaMAX supplemented with 2% B27, 1% P/S, 10 ng/ml EGF, 5 ng/ml FGF-2) was added before mechanical dissociation. The cells were cultured under standard cell culture conditions in a saturated atmosphere containing 21% O_2_ and 5% CO_2_ in floating conditions to allow the formation of neurospheres. The latter were passaged when they reached a diameter of approximately 0.05–0.1 mm. Cells were spun down at 1,200 rpm for 5 min and resuspended in 1 ml fresh culture medium until single cell suspension. Neurospheres where then distributed over new T25 flasks. Passages 3–6 were used for *in vitro* experiments.

### Cell Culture and Coating Conditions

P24 plates were coated overnight with PDL or PornT at concentrations of 10, 50, and 100 μg/ml for each coating medium. Mixed groups contained either 5 μg/ml PDL and 5 μg/ml PornT (“5+5”) or 10 μg/ml PDL and 10 μg/ml PornT (“10+10”). Neurospheres from passage 3–6 were seeded at a density of 50 spheres/well. Seeding was done in culture media devoid of growth factors. For immunocytochemistry and migration assays, the neurospheres were plated on glass coverslips coated as indicated before, whereas for protein extraction experiments the neurospheres were directly seeded onto P24 plates. Cells were allowed to differentiate for 24 h. For protein extractions, neurospheres were washed once with D-PBS before adding 30 μl of lysis buffer (NP-40, 2% protease inhibitor, 1% phosphatase inhibitor cocktail; Roche) per well. Neurospheres were scratched from the well and the solution was incubated for 15 min on ice. The solution was then centrifuged at 14,000 rpm for 15 min and the supernatant was used for protein measurement. For immunocytochemistry, neurospheres were washed with D-PBS and fixed on coverslips by incubation with 4% paraformaldehyde (PFA) for 15 min and immediately treated for immunocytochemistry.

### Bradford Protein Assay and Western Blotting

Protein concentration was determined by a Bio-Rad protein assay kit (Bio-Rad #500-0116) according to the manufacturer’s protocol. 10 μg of protein were fractioned by SDS-PAGE and transferred to a nitrocellulose blotting membrane (GE Healthcare, Germany). The membrane was washed three times with 1x Tris buffered saline with Tween20 (TBST) and incubated with 5% non-fat dry milk in TBST for 60 min. After three more washing steps with TBST, the membrane was incubated with rabbit anti-nestin (1:500, Abcam, Germany), rabbit anti-β-actin (1:2,000, EMD Millipore, Germany), rat anti-GFAP (1:1000, Invitrogen; Carlsbad, CA, USA), rabbit anti-p-AKT (1:2,000, EMD Millipore)and rabbit anti-Akt (1:2,000, EMD Millipore) at 4°C overnight. Membranes were washed three times for 10 min and incubated with a 1:5,000 dilution of anti-rat or anti-rabbit horseradish peroxidase-conjugated antibodies (Santa Cruz Biotechnology, Dallas, TX, USA) for 1 h at room temperature. Blots were washed with TBST three times and developed with the ECL system (GE Healthcare; Germany) according to the manufacturer’s protocols. Photos were taken with Fusion FX7 (Peqlab) and analyzed with ImageJ. Samples were blinded to experimenters and analysts.

### Immunocytochemistry

Coverslips were washed three times with PBS with 0.1% triton (PBS-T) for 10 min and incubated with 5% normal donkey serum (NDS) in PBS-T and washed again three times with PBS-T. Primary antibodies rabbit anti-nestin (1:1,000) and rat anti-GFAP (1:1,000) were incubated overnight at 4°C. After three more washing steps secondary antibodies (donkey anti-rabbit 488; donkey anti-rat 594; Invitrogen, Portland, OR, USA) were incubated at a concentration of 1:1,500 for 1 h at room temperature. Following three more washing steps, coverslips were mounted with vectashield mounting medium with DAPI (Vector, USA). After staining, photos were taken with an Olympus BX51 upright microscope and analyzed with ImageJ to determine the length of neurite-like branches using the NeuronJ plugin. Samples were blinded to experimenters and analysts.

### Neurosphere Migration Assay

Neurospheres in the range of 100–200 μm were seeded on coverslips coated with as before to evaluate the influence of coating concentration on expansion of the neurosphere as a measurement of migration. Alternatively, fixed concentrations of 50 μg/ml of either PDL or PornT was used to evaluate the response of the spheres to proteins known to influence the migration and differentiation of NPCs depending on the coating of choice. In the latter case neurospheres were stimulated with 500 ng/μl of stromal-cell derived factor-1alpha (SDF-1a, R&D systems, Germany) or 50 IU/ml of rhEpo (NeoRecormon, Roche, Switzerland). Expansion was tracked for 8 h on a Zeiss inverted microscope equipped with a cell culture chamber. Images were taken every 10 min with a 10x phase contrast objective. Expansion of the spheres was analyzed using custom designed macros on ImageJ. The increase in area occupied by the neurosphere was calculated as $r\,A=\frac{f\,A}{i\,A}$, where, *rA* is the ratio of the sphere area, *fA* is the area at the end of the recording, and *iA* is the area at the beginning of the recording. Data is represented as ratio of the respective control (**Figures 7** and **8**) or as rA (Supplementary Figure S1). At least 10 neurospheres were tracked in a total of 3–4 experiments per evaluated condition.

### Statistics

Group comparisons were done by a one-way ANOVA with subsequent Bonferroni correction. Simple pairwise comparisons were performed using Mann–Whitney using Sigma-Plot. Data are presented as mean ± SD and a *P*-values lower than 0.05 were considered significant.

## Results

**FIGURE 1:**
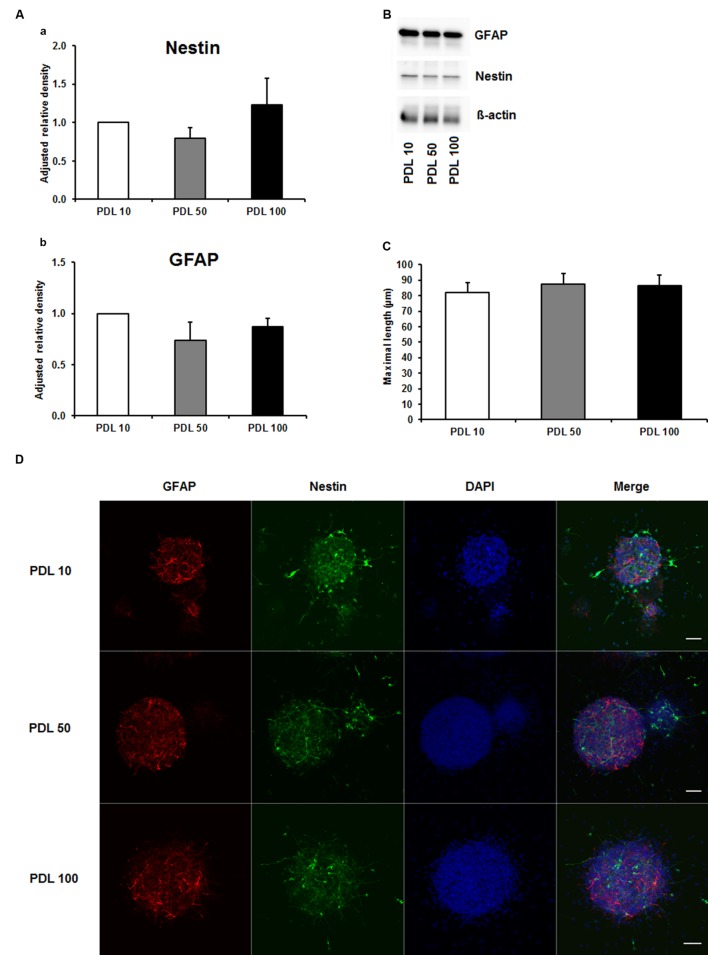
**Effects of increased concentrations of poly-D-lysine on neurosphere maturation after 24 h. (A)** Western blot analysis of (a) nestin (177 kDa) and (b) GFAP (48 kDa) protein expression (*n* = 4); data were normalized to β-actin and PDL 10 served as control group. **(B)** Representative Western blots of GFAP, nestin and β-actin (45 kDa). **(C)** Statistical analysis of neuronal branch length measurements **(D)**. Representative confocal images of neurospheres after 24 h of cultivation stained for GFAP (red), nestin (green), and DAPI (blue); Space bar equals 50 μm. PDL 10: 10 μg/ml; PDL 50: 50 μg/ml; PDL 100: 100 μg/ml. All data represent mean ± standard deviation.

To investigate the aforementioned lack of consensus on whether to use PDL or PornT for *in vitro* studies, we first tested increasing concentrations of both compounds by means of neurosphere differentiation assays after 24 h of incubation under standard cell culture conditions. No significant difference in the amount of GFAP and nestin expression was found between increasing concentrations of PDL conditions in the performed Western blots (**Figures 1A,B**). Furthermore, morphological analysis of differentiated neuroblasts did not reveal statistical differences as indicated (**Figures 1C,D**).

**FIGURE 2:**
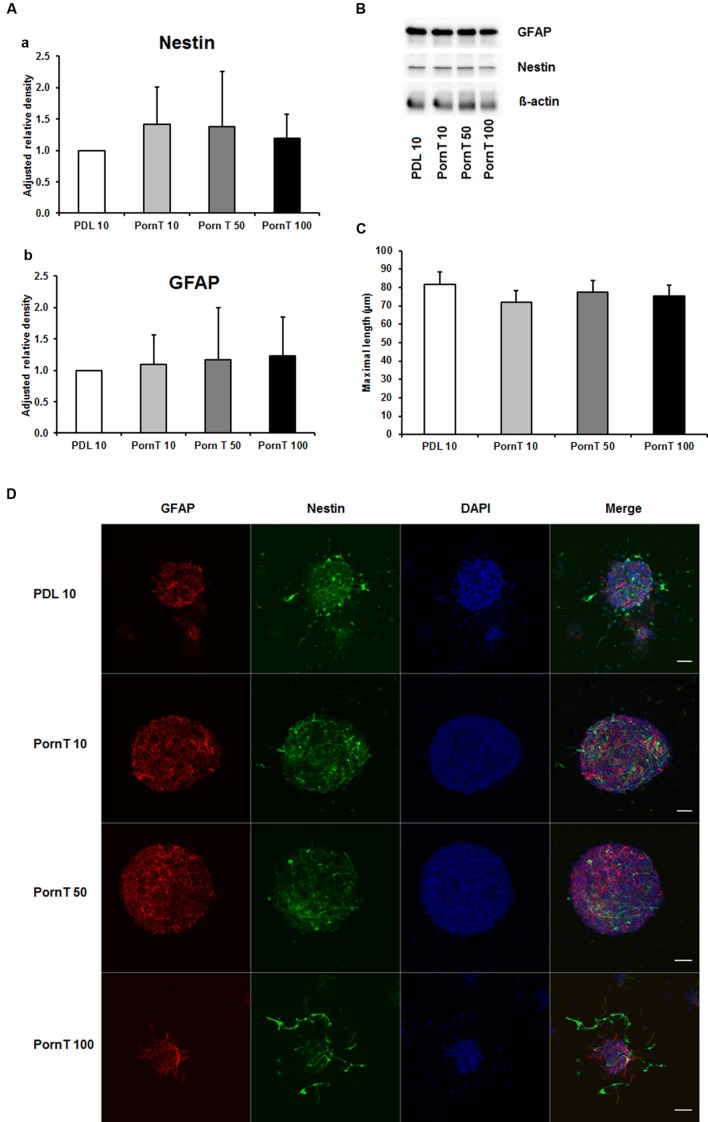
**Effects of increased concentrations of polyornithine on neurosphere maturation after 24 h. (A)** Western blot analysis of (a) nestin (177 kDa) and (b) GFAP (48 kDa) protein expression (*n* = 4); data were normalized to β-actin and PDL 10 served as control group. **(B)** Representative Western blots of GFAP, nestin, and β-actin (45 kDa). **(C)** Statistical analysis of neuronal branch length measurements **(D)**. Representative confocal images of neurospheres after 24 h of cultivation stained for GFAP (red), nestin (green), and DAPI (blue); Space bar equals 50 μm. PornT 10: 10 μg/ml; PornT 50: 50 μg/ml; PornT 100: 100 μg/ml. All data represent mean ± standard deviation.

Since numerous papers also used PornT as coating substance in their *in vitro* or *ex vivo* experiments (Ray et al., 1995; Wachs et al., 2003; Oudin et al., 2011; Ladiwala et al., 2012; Sonego et al., 2015), we next examined increasing concentrations of PornT under the same conditions used for PDL. The PDL 10 groups served as control group as our previous performed experiments already revealed that there is no significant difference in increasing PDL concentrations on neurosphere differentiation. All three tested concentrations of PornT did not show significant differences in the protein amount of GFAP and nestin as tested by means of Western blotting (**Figures 2A,B**). When compared to the PDL 10 groups, none of the groups varied statistically from each other (**Figures 2A,B**). Again, a morphological analysis of differentiated neuroblasts such as length of branches did not reveal statistical differences as already shown above for PDL (**Figures 2C,D**).

**FIGURE 3:**
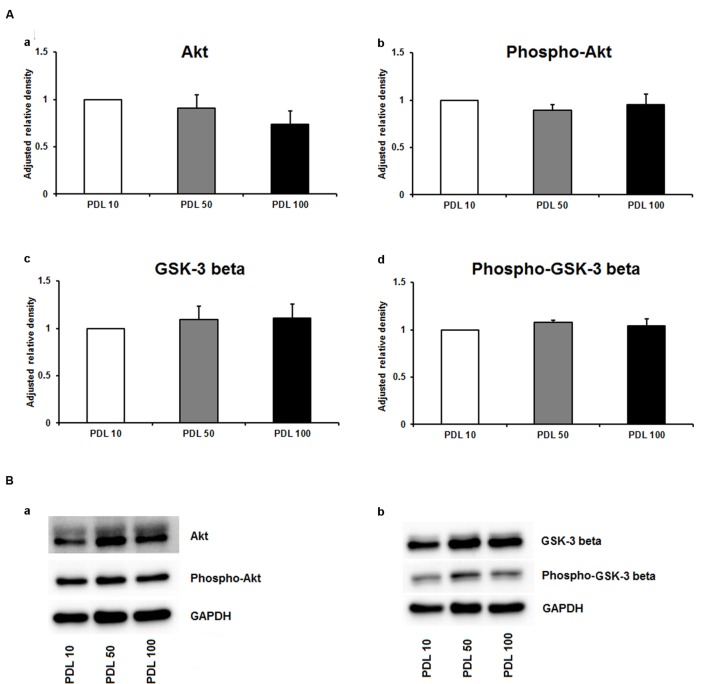
**Protein expression of intracellular proteins after 24 h cultivation of neurospheres on different coating concentrations of poly-D-lysine. (A)** Western blot analysis of (a) Akt, (b) pAkt (c) GSK-3 beta, and (d) phopho-GSK-3 beta (*n* = 4); data were normalized to GAPDH and PDL 10 served as control group. **(B)** Representative Western blots of (a) Akt, pAkt and (b) GSK-3 beta, phopho-GSK-3 beta, and GAPDH. All data represent mean ± standard deviation.

**FIGURE 4:**
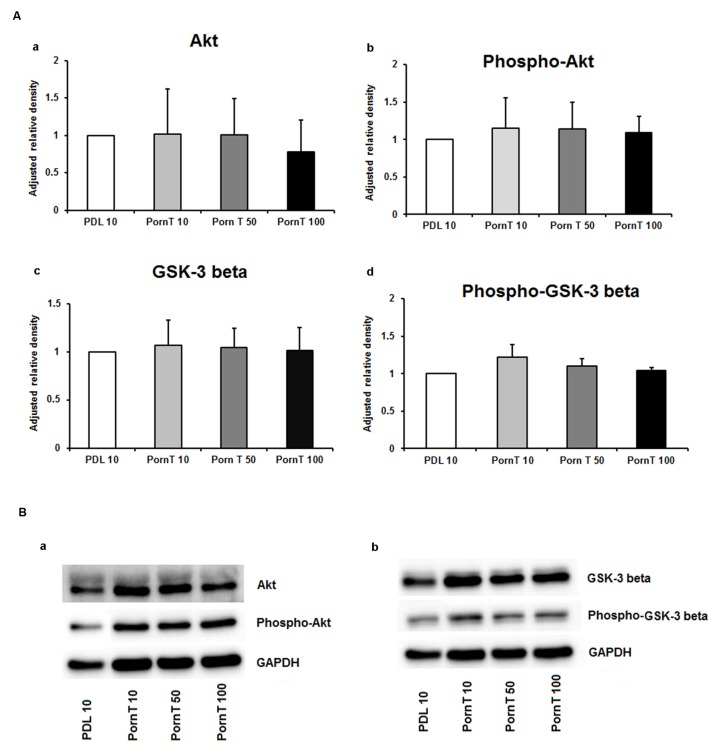
**Protein expression of intracellular proteins after 24 h cultivation of neurospheres on different coating concentrations of polyornithine. (A)** Western blot analysis of (a) Akt, (b) pAkt (c) GSK-3 beta, and (d) phopho-GSK-3 beta (*n* = 4); data were normalized to GAPDH and PDL 10 served as control group. **(B)** Representative Western blots of (a) Akt, pAKT and (b) GSK-3 beta, phopho-GSK-3 beta, and GAPDH. All data represent mean ± standard deviation.

Since neuronal growth and differentiation is regulated by a number of intracellular mechanisms (Shioda et al., 2009), we next investigated proteins that play a crucial role in these underlying endogenous mechanisms. Akt, pAkt, GSK-3-beta, and pGSK-3-beta were further examined by means of Western blotting. For the three increasing PDL concentrations, neither of the tested proteins showed differences in the performed Western blots (**Figures 3Aa**–**d,Ba,b**). Additional Western blots with increasing amounts of PornT again did not reveal significant differences in the amount of Akt, pAkt, GSK-3-beta, and pGSK-3-beta (**Figures 4Aa**–**d,Ba,b**).

Given that neither increasing concentrations of PDL nor increasing concentrations of PornT could reveal differences in neurosphere differentiation patterns, we further investigated if a mixture of PDL + PornT would be preferable and could thus have an effect on the differentiation pattern of neurospheres. As such, 5 μg/ml PDL + 5 μg/ml PornT or 10 μg/ml PDL + 10 μg/ml PornT were tested as possible coating concentrations. The 5+5 groups was chosen as 10 μg/ml was the minimal dose for both PDL and PornT in all the aforementioned experiments.

**FIGURE 5:**
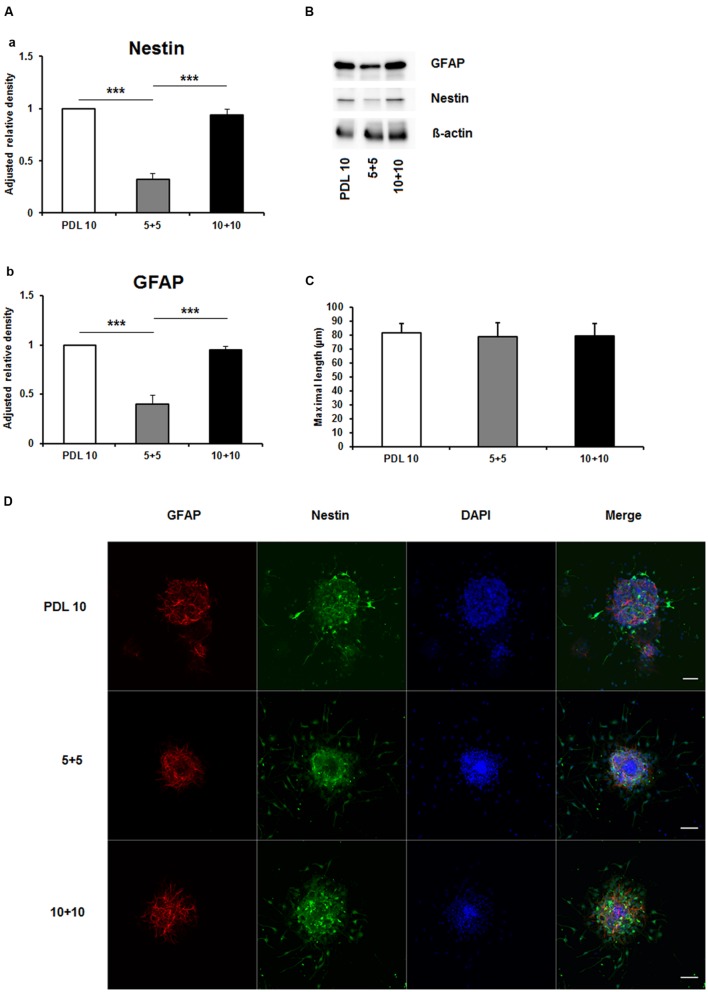
**Effects of increased concentrations of mixed concentrations of poly-D-lysine and polyornithine on neurosphere maturation after 24 h. (A)** Western blot analysis of (a) Nestin (177 kDa) and (b) GFAP (48 kDa) protein expression (*n* = 4); data were normalized to β-actin and PDL 10 served as control group. **(B)** Representative Western blots of GFAP, nestin and β-actin (45 kDa). **(C)** Statistical analysis of neuronal branch length measurements **(D)**. Representative confocal images of neurospheres after 24 h of cultivation stained for GFAP (red), nestin (green), and DAPI (blue); Space bar equals 50 μm. 5+5: 5 μg/ml PDL+ 5 μg/ml PornT; 10+10: 10 μg/ml PDL + 10 μg/ml PornT. All data represent mean ± standard deviation. ^∗∗∗^*P* ≤ 0.001.

**FIGURE 6:**
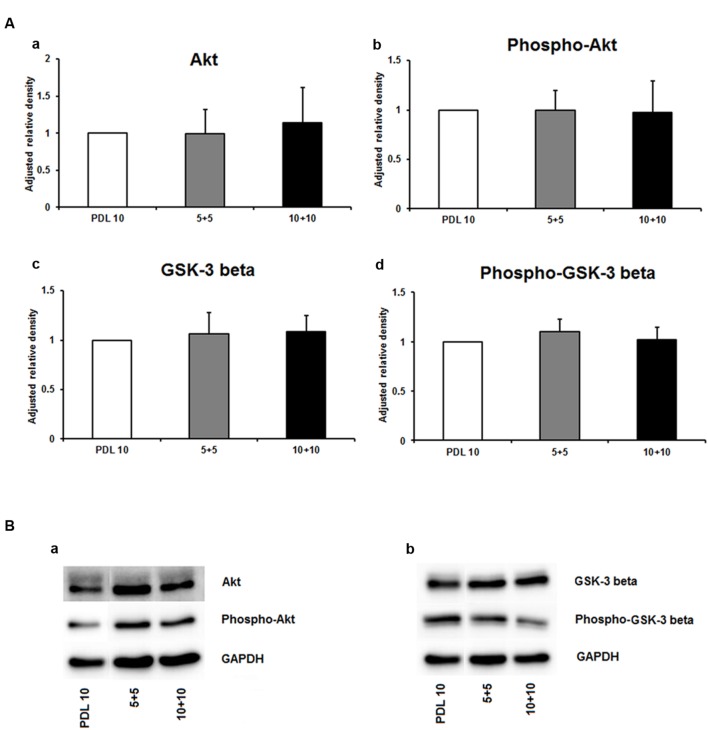
**Protein expression of intracellular proteins after 24 h cultivation of neurospheres on different coating concentrations of poly-D-lysine + polyornithine. (A)** Western blot analysis of (a) Akt, (b) pAKT (c) GSK-3 beta, and (d) phopho-GSK-3 beta (*n* = 4); data were normalized to GAPDH and PDL 10 served as control group. **(B)** Representative Western blots of (a) Akt, pAKT and (b) GSK-3 beta, phopho-GSK-3 beta, and GAPDH. All data represent mean ± standard deviation.

As depicted in **Figure 5A**, the protein amount of both nestin and GFAP were significantly decreased by more than 50% in the 5+5 groups when compared to both the PDL 10 and the 10+10 groups (*P* < 0.001). No differences were noticed in protein load as indicated by b-actin signal (**Figure 5B**). However, when statistical analysis of neuronal branch length was performed no significant difference was found between the three groups (**Figures 5C,D**). Consequently, the four intracellular proteins Akt, pAkt, GSK-3-beta, and pGSK-3-beta were also investigated for the 5+5 and 10+10 groups. However, these additional Western blots did not show statistical differences (**Figure 6**) in protein expression and phosphorylation levels.

**FIGURE 7:**
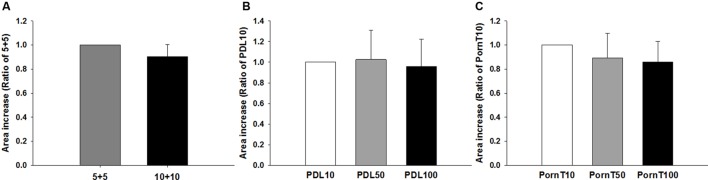
**Increase of area of the neurosphere is independent of coating condition**. Cells emerging from the neurospheres occupy the same area 8 h after seeded on the **(A)** 5+5 or 10+10 condition, **(B)** on increasing concentrations of PDL or **(C)** on increasing concentrations of PornT. 5+5: 5 μg/ml PDL+ 5 μg/ml PornT; 10+10: 10 μg/ml PDL + 10 μg/ml PornT. *rA* data was normalized to its respective experimental control and presented as mean ± standard deviation (*n* = 3 experiments).

Having found no differences in morphological or molecular markers related to differentiation, we then asked whether different coating conditions would affect migration of cells. To this end, neurospheres were seeded on coverslips coated identically as in previous experiments. As depicted in **Figure 7**, no differences were found between the 5+5 and 10+10 conditions (**Figure 7A**) or with increasing concentrations of PDL or PornT (**Figures 7B,C**). All neurospheres increased their area in a linear manner between 2 and 2.5 times with relation to their original size regardless of the coating condition (Supplementary Figures S1 and S2). This is consistent with the absence of changes in expression of pAkt and pGSK-3-beta between the different conditions.

**FIGURE 8:**
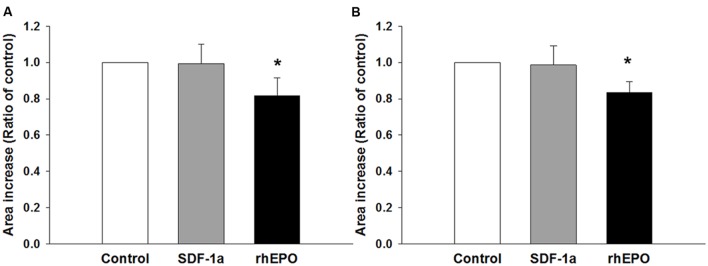
**Neurospheres respond in similar ways to chemical interventions regardless of the coating condition**. SDF-1a did not promote migration of cells whereas rhEpo reduced migration by 20% when the neurospheres were seeded on **(A)** 50 μg/mL of PDL and **(B)** 50 μg/mL of PornT. *rA* data was normalized to its respective experimental control and presented as mean ± standard deviation (*n* = 4 experiments). ^∗^*P* < 0.05.

Finally, we wondered whether or not the coating condition would have any effect on the response of the neurospheres to stimulation with agents that have been proven to stimulate NPC migration and differentiation. To this end, the neurospheres were seeded as before but on a fixed concentration of either PDL or PornT (50 mg/ml each). Expansion of the neurospheres was recorded and analyzed as before. Migration results showed that SDF-1a had no effect on the increase of area whereas rhEpo caused a surprising reduction in area increase of approximately 20% when expressed as a ratio of the control condition [rhEPO on PDL = 0.81 ± 0.096 (*P* < 0.05); rhEPO on PornT = 0.83 ± 0.058 (*P* < 0.05); **Figures 8A,B**]. This is in contrast with Robin et al. (2006) and Wang et al. (2006) who found an induction of NPC migration after treatment with similar doses of SDF-1a and rhEpo. However, in both cases neurospheres were seeded either on brain slices or monolayers of mouse brain endothelial cells, which might have provided additional signaling in response to treatments, and length of radial processes was quantified rather than increment of area occupied by cells emerging from the neurosphere.

## Discussion

*In vitro* and *ex vivo* cultivation of stem cells and NPCs alike are essential methods for testing characteristic behavior of these cells under various experimental settings. For the study of differentiation, survival, maturation, and migration of cultivated progenitor cells, attachment to synthetic coating compounds is inevitable for the success of these experiments. PDL and PornT are the two most commonly used coating compounds to ensure better attachment of cells to a preferred underground (Greilberger et al., 1997; Ramsay and Gumbleton, 2002; Louis and Reynolds, 2005; Lai et al., 2008; Ahlenius et al., 2009). However, there is no broad consensus as to which of these two synthetic amino acid chains is favorable for the underlying processes of cell actions. Furthermore, different laboratories use different concentrations, which makes it even harder to agree on a standard protocol for *in vitro* and *ex vivo* experiments. Therefore, this study aimed at investigating if increasing coating concentrations of PDL and PornT alter differentiation or maturation of neurospheres after 24 h of cultivation.

Neither PDL nor PornT alone showed differences in the protein expression of nestin and GFAP in neurospheres after 24 h of culture. GFAP serves as astrocytic marker, whereas nestin marks NPCs. The maturation of neurospheres with respect to neuroblast and astrocyte development did not differ under the increasing concentrations of PDL or PornT alone. The combined use of PDL + PornT in a concentration of 5 μg/ml + 5 μg/ml showed a significant decrease in the amount of both nestin and GFAP protein expression. A decrease in GFAP expression could indicate that less neuronal precursor cells develop an astrocytic phenotype. A decrease in the amount of nestin could indicate that more NPCs develop into neuroblasts, oligodendrocytes, or astrocytes. However, significantly less GFAP expression detected in these samples already indicated that the shift does not go into the direction of astrocytes. Furthermore, the cultivation time of 24 h is too short for the differentiation into neurons or oligodendrocytes. As both GFAP and nestin expression are significantly decreased it might be the case that neurospheres lose their NPC cell specific phenotype when cultivated with an insufficient amount of PDL or PornT. Another possible explanation could be an increase in cell death. Therefore, additional proteins involved in apoptotic pathway should be investigated to further elucidate whether an insufficient amount of coating material results in increased cell death.

To further study differentiation patterns, intracellular proteins investigated were Akt, pAkt, GSK-3-beta, and pGSK-3-beta. All four proteins are crucially linked and together regulate neurogenesis, cell proliferation, and cell growth (Endo et al., 2006; Peltier et al., 2007; Chojnacki and Weiss, 2008; Hur and Zhou, 2010; Kitagishi et al., 2012). Akt is activated due to phosphorylation. Interestingly for this study was the ability of pAkt to regulate neuronal differentiation and maturation. This is achieved in part through the ability of pAkt to phosphorylate GSK-3-beta thereby inhibiting its suppressive effect on β-catenin. An increase in pGSK-3-beta could thus indicate an increase in neurogenesis and neuronal differentiation. Another possible pathway for induced cell proliferation and maturation is the activation of downstream targets of pAkt like mTOR and VEGF. However, neither Akt nor GSK-3-beta did show significant differences in their phosphorylation pattern in cultivated neurospheres. As we could not find significant differences in the expression profiles of all four proteins in neurospheres tested under all aforementioned conditions this supports the conclusion that there is no difference regarding molecular pathways of neurogenesis and differentiation depending on the coating conditions chosen. However, due to decreased GFAP and nestin expression in the 5+5 groups, we rather conclude that this group shows limitations in maintaining NPC specific cell type markers. Proteins involved in apoptotic pathways remain to be investigated to test this possibility.

None of the tested conditions, 5+5, 10+10 or PDL/PornT 10, 50, 100 mg/ml affected expansion of the area occupied by neurospheres. Interestingly SDF-1a had no effect on migration of cells, whereas we detected a reduction of 20% of migration when spheres were stimulated with rhEpo regardless of the coating condition. The lack of effect of SDF-1a and the negative response to rhEpo most likely reflects the lack of trophic support found in other culture systems that incorporate tridimensional features like seeding over monolayers of other cells or organotypic slices in which the chemical treatment might enhance already existing interactions between the NPCs and their surroundings. Indeed, rhEpo promoted matrix metalloproteinase 2 release from endothelial cells, thus promoting NPC migration (Wang et al., 2006), which could explain the negative effect seen here as neurospheres contain mostly GFAP expressing cells, few neuroblasts and no endothelial cells (Sanchez-Mendoza et al., 2013). We can then conclude that the coating condition has no influence on migration patterns of NPCs when these are stimulated with different chemical interventions.

In conclusion, both PDL and PornT can be used to cultivate cells as both coating materials achieve equivalent results. Nevertheless, when using only one compound, we propose a minimal dosage of 10 μg/ml of either PDL or PornT as our results indicate that the differentiation pattern of neurospheres is significantly altered when only 5 μg/ml of one compound is used.

## Author Contributions

ES-M: performed research, study design, wrote the manuscript; JS: performed research, study design, wrote the manuscript; DH: wrote the manuscript; TD: study design, wrote the manuscript.

## Conflict of Interest Statement

The authors declare that the research was conducted in the absence of any commercial or financial relationships that could be construed as a potential conflict of interest.
