# Supplementary material for: Characterization of Seeding Conditions for Studies on Differentiation Patterns of Subventricular Zone Derived Neurospheres
Source: Front Cell Neurosci. 2016 Mar 7;10:55. doi: 10.3389/fncel.2016.00055 (PMC4779939; doi:10.3389/fncel.2016.00055)
Supplement: Supplementary file 1 [file Image_1.PDF]

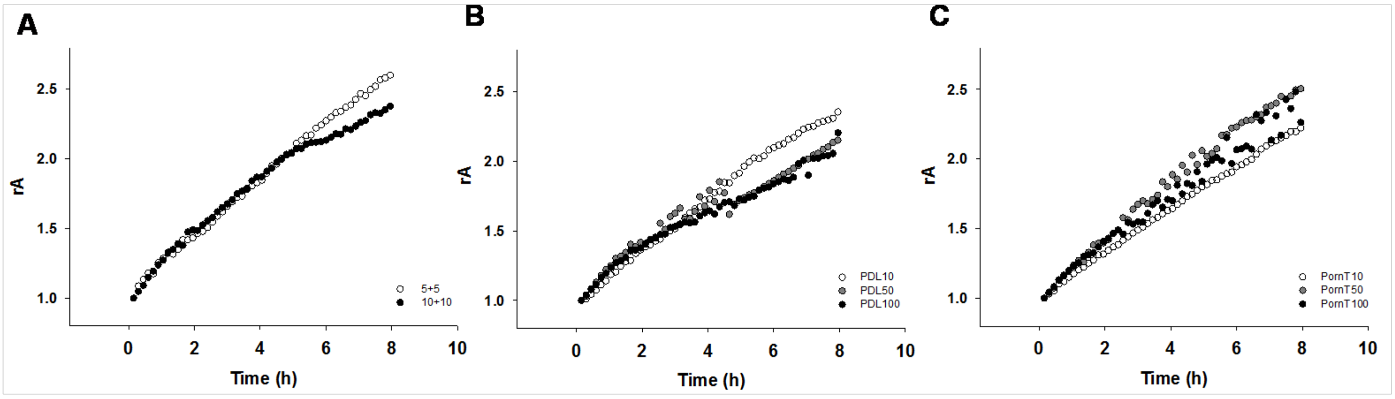

Supplementary Figure 1. Measurement of area increase (*rA*). *rA* data show a linear increase in the area occupied by the neurospheres during a period of 8 h when they are seeded on (A) 5+5 or on 10+10 condition, (B) on increasing concentrations of PDL or (C) on increasing concentrations of PornT. 5+5: 5 µg/ml PDL+ 5 µg/ml PornT; 10+10: 10 µg/ml PDL + 10 µg/ml PornT. Despite some differences seen at late time points (i.e., >5h), these are not statistically significant. Data represents mean values obtained from 3 experiments. Standard deviation has been omitted for clarity.
