# Supplementary material for: Characterization of Seeding Conditions for Studies on Differentiation Patterns of Subventricular Zone Derived Neurospheres
Source: Front Cell Neurosci. 2016 Mar 7;10:55. doi: 10.3389/fncel.2016.00055 (PMC4779939; doi:10.3389/fncel.2016.00055)
Supplement: Supplementary file 2 [file Image_2.PDF]

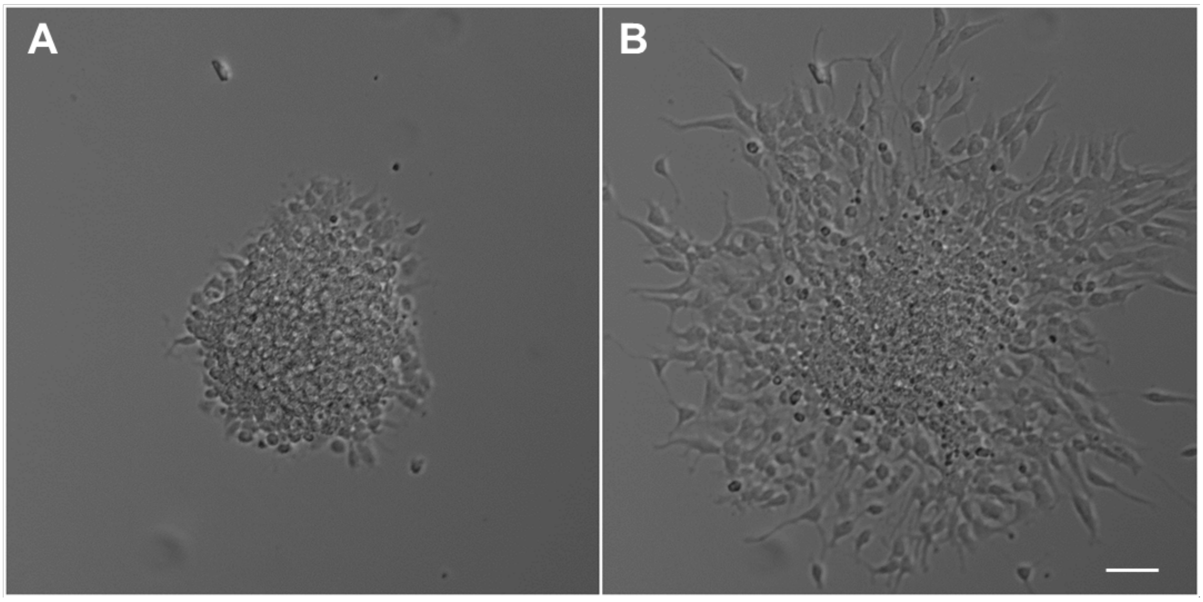

Supplementary Figure 2. Representative image of neurosphere growth at (A) 10 min after initiation of recording and at 8 h (B). Areas were calculated using custom macros developed for ImageJ. Scale bar: 50 µm.
